# Hepatic Failure and Hyperbilirubinemia Secondary to Hemolytic Disease of the Newborn

**DOI:** 10.1155/2024/9180404

**Published:** 2024-08-20

**Authors:** Matthew Reinhardt, Marya L. Strand, Dajana Sabic

**Affiliations:** ^1^ Department of Pediatrics Saint Louis University School of Medicine, St. Louis, MO, USA; ^2^ Department of Neonatal-Perinatal Medicine Akron Children's Hospital, Akron, OH, USA; ^3^ Department of Pediatrics Baylor College of Medicine, Houston, TX, USA

## Abstract

Anti-D antibody is the most common cause of hemolytic disease of the newborn (HDN). Other antigens have emerged, causing significant damage to the newborn. We report a case of a 31-week dichorionic diamniotic twin born emergently to a mother with anti-C and anti-D antibodies who required multiple percutaneous umbilical cord blood sampling (PUBS) and transfusions. Extensive resuscitation at birth was required due to severe anemia, hypovolemia, and prematurity. Ensuing liver damage occurred with subsequent profound coagulopathy, transaminitis, and direct hyperbilirubinemia that required significant support. This patient developed several unique findings including the inability to monitor oxygen saturations due to dermal bilirubin deposits and staining of baby teeth. This case report highlights the importance of early identification of additional Rh antibodies due to concern for development of more severe forms of HDN.

## 1. Introduction

Rhesus (Rh) antigens are transmembrane proteins expressed on erythrocytes, transporting certain molecules across the erythrocyte's cell membrane [1, 2]. Over 50 different types of Rh antigens exist, most commonly D, c, E, C, and e (listed from most to least immunogenic) [3, 4]. D antigen is most often seen with hemolytic disease of the newborn (HDN). The most common non-D antigens are E, Kell, and C (listed most to least common) [5], although Kell is not in the Rh family. The remaining antigens can be expressed individually or in pairs (ce, Ce, cE, or CE) [4]. D antigen is responsible for 50% of maternal alloimmunization [5]. HDN severity depends on which Rh antigen triggers the isoimmunization. Anti-c antibody and the combination of anti-c and anti-E antibodies can generate a severe form of HDN [5].

For HDN to occur, a Rh-negative mother must carry a Rh-positive fetus. The mother then requires exposure to the fetus' Rh-positive erythrocytes to trigger sensitization and for the mother to form anti-Rh IgG. This can be due to fetal-maternal hemorrhage during labor, abortion, placenta previa, chorionic villus sampling, amniocentesis, Cesarean delivery, or any form of uterine/vascular trauma. Subsequent pregnancies are at risk for isoimmunization due to maternal anti-Rh IgG crossing the placenta and binding the fetal erythrocytes. HDN can also be caused by ABO incompatibility, although it generally causes less severe cases of HDN [3, 6]. This case report describes a severe presentation of HDN that led to unique sequelae.

## 2. Case Presentation

A 1635-gram twin male (twin A) was born by emergent Cesarean section at 31 weeks 3 days to a 36-year-old gravida 4 para 3 mother due to bleeding and fetal bradycardia following a percutaneous umbilical cord blood sampling (PUBS) procedure. The pregnancy was complicated by dichorionic diamniotic twin pregnancy, a known history of anti-C and anti-D antibodies requiring multiple PUBS procedures during this gestation, and a previous history of drug use and active tobacco use. Maternal antibody titers were tracked during the pregnancy (Table 1). The mother received good prenatal care with normal serologies, excluding unknown Group B *Streptococcus* status and O-negative blood type.

Due to anti-C and anti-D antibodies, this mother underwent routine middle cerebral arterial Doppler measurements during pregnancy. At 30 weeks 4 days gestation, the mother underwent three PUBS procedures over a one-week period. Each twin received three PRBC transfusions; one with each PUBS. Before the third transfusion both twins had hemoglobin's of 3.7 g/dL. During the third transfusion, Twin A lost access, developed bleeding, became bradycardic, and both infants were delivered via emergent Cesarean section.

At delivery, Twin A exhibited poor tone, bradycardia, apnea, and pallor. He was emergently intubated, ventilated, and received chest compressions. An emergent umbilical venous catheter was placed. Packed red blood cells, platelets, fresh frozen plasma, and factor VII were given in the delivery room with additional transfusions later in the NICU. Apgar scores were 0, 2, and 4 at 1, 5, and 10 minutes of life, respectively. Exam revealed significant hepatomegaly 8 centimeters below the costal margin, abdominal petechiae, delayed capillary refill, and bleeding from the nares and umbilical line consistent with disseminated intravascular coagulation.

At admission, twin A had a hemoglobin of 12 g/dL. He received four subsequent PRBC transfusions due to ongoing hemolysis during his course. Thrombocytopenia, hypofibrinogenemia, and an elevated international normalized ratio (INR) were also present (platelet nadir was 9 × 10^9^/L, maximum INR was 7.3, and minimum fibrinogen level was <70 mg/dL). Additional Vitamin K, Factor VII, cryoprecipitate, platelet, and fresh frozen plasma transfusions were administered.

**Figure 1:**
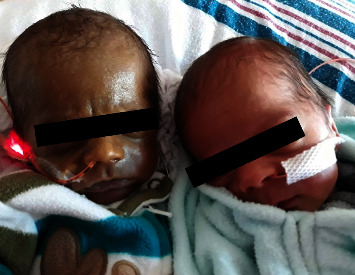
Twin A and twin B side-by-side.

Twin A had signs of liver injury, with a peak ALT and AST of 561 IU/L and 2,531 IU/L, respectively. Initial total bilirubin level at 4 hours was 4.2 mg/dL, increasing to 9.4 mg/dL by 36 hours with direct and indirect bilirubin levels of 1.2 mg/dL and 8.2 mg/dL, respectively. Peak total, direct, and indirect bilirubin levels were 56 mg/dL, 43 mg/dL, and 17.3 mg/dL, causing significant skin discoloration (Figure 1). Treatment for hyperbilirubinemia included one month of ursodiol for the direct hyperbilirubinemia and 15 days of intensive phototherapy for indirect hyperbilirubinemia. Pediatric hematology was consulted for the hyperferritinemia, and decision was made to not use chelation therapy. Other etiologies of liver damage were ruled out, including infectious, metabolic, and genetic causes. Throughout the remaining hospital course, the hepatomegaly persisted but the synthetic function of the liver recovered. Markers of cell destruction revealed serum ferritin peaking at 18,138 ng/mL and alkaline phosphatase at 294 *µ*g/dL. Abdominal ultrasound showed no evidence of anatomical abnormalities in the liver or gallbladder. Total parenteral nutrition was initiated after delivery and full enteral feeds were reached by day 20.

Additional complications included respiratory distress requiring intubation at delivery, surfactant administration, and mechanical ventilation. Extubation to CPAP was achieved by day 12 but he was reintubated on day 17 for perceived hypoxemia. Despite escalating support (100% FiO_2_ and inhaled nitric oxide at 20 parts per million), his saturations remained around 80%. A concurrent blood gas demonstrated a PaO_2_ of >120 mmHg. When the pulse oximeter probe was moved to his ear lobe, the reading was consistently 98–100%. He was subsequently extubated to CPAP on day 17 and weaned to room air by day 35. The earlobe was the only location where an accurate oxygen saturation could be obtained. We hypothesize the density of bilirubin deposition in his skin prevented accurate reading of his oxygen saturation. Etiology of the respiratory distress was secondary to surfactant deficiency, complicated by pulmonary hemorrhage and pulmonary hypertension confirmed by echocardiography.

**Figure 2:**
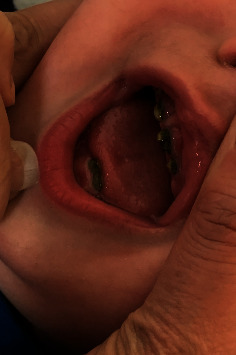
Bilirubin-stained teeth of patient at follow up appointment.

Due to his clinical course, a head ultrasound was obtained at 14 days, revealing a left grade two germinal matrix hemorrhage. A follow up brain MRI confirmed a prior germinal matrix hemorrhage. At outpatient follow ups, he demonstrated appropriate development and there are no current concerns for long-term liver or neurological damage. One prominent consequence of the hyperbilirubinemia is the discoloration of his teeth (Figure 2).

Twin B's course was significantly less complex, requiring one postnatal PRBC transfusion. He also received intravenous immunoglobulin (IVIG), albumin, and intensive phototherapy for hyperbilirubinemia.

Both infants were followed in the multidisciplinary neurodevelopmental clinic at 4, 8, and 12 months of corrected age with no noted deficits. Both infants received a Bayley Scale of Infant Development (3rd edition) at 18 months of corrected age for developmental evaluation, which revealed no delays and did not require subsequent evaluations. There are no current signs of bilirubin-induced neurologic dysfunction (BIND) in the infants. The infants were otherwise followed by their pediatrician. Written consent for reporting this case was obtained from the mother of the twins.

## 3. Discussion

In HDN, the neonate's erythrocytes are destroyed through extravascular hemolysis due to the binding of maternal anti-Rh IgG to the fetal or neonatal Rh antigens. The rate of hemolysis determines severity of clinical presentation. In mild cases, anemia and jaundice are well tolerated and resolve without treatment. In moderate and severe cases, indirect bilirubin can rise high enough to cause kernicterus. In severe cases, hydrops fetalis can develop, which can be fatal [7]. Elevated serum iron levels can also be seen secondary to HDN and/or multiple PRBC transfusions, leading to the deposition of iron in various organs, including the liver. Sequelae include coagulation dysfunction, hypoalbuminemia, and eventually liver failure [8–11]. Increased erythropoiesis and suppression of other cell lines in severe cases of HDN can lead to thrombocytopenia and leukopenia [10]. Recurrent in utero red cell transfusions also pose additional risk and have been associated with an increased risk of preterm labor, premature rupture of membranes, chorioamnionitis, and heart failure secondary to volume overload [12]. This patient's severe liver injury could be attributed to multiple mechanisms: hemolyzed erythrocytes adhering to hepatic vascular endothelium or hepatic macrophages, or occluding hepatic sinusoids [7]; anemia causing ischemic damage to hepatocytes; the dramatic increase in bilirubin could have led to cholestasis and subsequent liver damage; or iron accumulation in the liver could have led to the hepatic dysfunction and cholestasis [10, 13]. Two risk factors for developing cholestasis in HDN are intrauterine transfusions and maternal D-, c-, or K antibodies in combination with other erythrocyte antibodies. This patient had both risk factors [11]. Severe anemia also led to extramedullary hematopoiesis, causing hepatomegaly [14].

Treatment for HDN is typically supportive but may include blood product transfusions, IVIG, and phototherapy [3]. Long-term outcomes depend on severity of hemolysis. The 2012 LOTUS study revealed that fetal alloimmune anemia treated with intrauterine transfusions led to a low incidence (4.8%) of neurodevelopmental impairment, defined as cerebral palsy, severe developmental delay, or bilateral deafness [15], further supporting the importance of early identification and intervention.

Isoimmunization from anti-C and anti-D antibodies individually typically causes mild to moderate HDN [5]. Das et al. and Howard et al. both demonstrated that HDN caused by anti-D and anti-C in combination led to moderate to severe HDN [16, 17]. It is unclear if the combination creates a synergistic effect. Limited case reports exist discussing HDN involving anti-C antibodies, as it is one of the least immunogenic Rh antigens [3, 4]. However, HDN that is caused by anti-C antibodies tends to be more severe. An important reason for early identification of anti-C antibody is to ensure that the neonate receives C-antigen-negative blood if transfusion is warranted [16].

## 4. Conclusion

Our case presents a patient with known isoimmunization in utero, resulting in early delivery and delivery room resuscitation. Extremely elevated indirect and direct bilirubin levels were likely secondary to red blood cell hemolysis and hepatic damage. Density of skin deposition of bilirubin resulted in the inability to monitor oxygen saturations in the typical manner, and the hyperbilirubinemia also caused discoloration of the patient's teeth. This case highlights the importance of early testing for isoimmunization, understanding which Rh antigens can trigger more severe isoimmunization, close monitoring of fetal hemoglobin levels, and prompt management to prevent long-term sequelae.

## Figures and Tables

**Table 1:** Maternal antibody titers.

Gestation	Anti-D	Anti-C	Anti-S
5 weeks	1 : 256	1 : 2	Not detected
27 weeks 5 days	>1 : 2048	1 : 4	Not detected
Postdelivery (32 weeks)	>1 : 1024	1 : 256	1 : 1

## Data Availability

The patient information and figures were obtained from patient data with guardian consent. There is no further data beyond the references included in the manuscript.

## Abbreviations

DIC: Disseminated intravascular coagulation
FFP: Fresh frozen plasma
HDN: Hemolytic disease of the newborn
INR: International normalized ratio
IVIG: Intravenous immunoglobulin
PUBS: Percutaneous umbilical cord blood sampling
PRBCs: Packed red blood cells.

## Additional Points

*Article Summary*. A severe case of Hemolytic Disease of the Newborn resulting in hepatic failure and other unique sequelae.
